# Supplementary material for: Identifying a predictive level of serum C-terminal telopeptide associated with a low risk of medication-related osteonecrosis of the jaw secondary to oral surgery: A systematic review and meta-analysis
Source: PLoS One. 2025 May 5;20(5):e0318260. doi: 10.1371/journal.pone.0318260 (PMC12052178; doi:10.1371/journal.pone.0318260)
Supplement: S1 Table — (DOCX) [file pone.0318260.s001.docx]

| **Authors** | **Year** | **Country** | **Study design** | **Total (n)** | **Patients with ONJ (n)** | **Controls (n)** | **Bisphosphonates indication** | **Route of administration** | **sCTX assay method** | **sCTX time of assessment** |
| --- | --- | --- | --- | --- | --- | --- | --- | --- | --- | --- |
| **Kwon Yong-Dae et al.** | 2009 | South Korea | Retrospective cohort | 18 | 18 | 0 | Osteoporosis | Oral | Unavailable | At the time of the ONJ |
| **Lazarovici et al.** | 2010 | Israel | Prospective cohort | 78 | 18 | 60 | Osteoporosis (n=51)  Cancer (n=27) | Oral (n=51)  IV (n=27) | ELISA | Prior to surgery |
| **Atalay et al.** | 2011 | Turkey | Retrospective cohort | 20 | 20 | 0 | Cancer | IV | ECLIA | Prior to surgery |
| **Kwon Yong-Dae et al** | 2011 | South Korea | Retrospective cohort | 84 | 23 | 61 | Osteoporosis | Oral | ECLIA | At the time of the ONJ |
| **Kwon Yong-Dae et al.** | 2012 | South Korea | Retrospective cohort | 6 | 6 | 0 | Osteoporosis | Oral | ECLIA | At the time of the ONJ |
| **Hutcheson et al.** | 2014 | Australia | Prospective cohort | 950 | 4 | 946 | Osteoporosis | Oral | ECLIA | Prior to surgery |
| **Kim et al.et al.** | 2018 | Republic of Korea | Case/control | 125 | 41 | 84 | Osteoporosis | Oral (n=102)  IV (n=23) | Unavailable | Prior to surgery |

Supplementary Table 1. Characteristics of the 7 included studies

n: number; ONJ : Osteonecrosis of the jaw ; IV : intravenous; sCTX : serum C-terminal telopeptide of type I collagen; ECLIA: Electrochemiluminescence immunoassay; ELISA: Enzyme-Linked Immunosorbent Assay.
