# Supplementary material for: Identifying a predictive level of serum C-terminal telopeptide associated with a low risk of medication-related osteonecrosis of the jaw secondary to oral surgery: A systematic review and meta-analysis
Source: PLoS One. 2025 May 5;20(5):e0318260. doi: 10.1371/journal.pone.0318260 (PMC12052178; doi:10.1371/journal.pone.0318260)
Supplement: S2 Table — (DOCX) [file pone.0318260.s002.docx]

Supplementary Table 2. Modified Newcastle-Ottawa Quality Assessment Scale (NOS) for Cohort Studies (Table 2a) and Case Control Studies (Table 2b) applied to the Studies included in the Meta-analysis.

Table 2a. NOS for the 6 Cohort Studies included.

|  | Selection ^†^ | | | |  | Outcome^††^ | | | |
| --- | --- | --- | --- | --- | --- | --- | --- | --- | --- |
| Included Studies | Representativeness of the exposed cohort | Selection of the Non-exposed Cohort | Ascertainment of Exposure | Incident Disease | Comparability^‡^ | Assessment of Outcome | Length of Follow-up | Adequacy of Follow-up | Total No. of stars |
| Kwon Yong-Dae et al. (2009) | A* | C | A* | B | C | B* | A* | D | 4 |
| Lazarovici et al.  (2010) | A* | A* | A* | A* | A** | B* | A* | D | 8 |
| Kwon Yong-Dae et al. (2011) | A* | B | A* | B | A* | B* | A* | D | 5 |
| Atalay et al. (2011) | A* | C | A* | B | C | B* | A* | D | 4 |
| Kwon Yong-Dae et al. (2012) | A* | C | A* | B | C | A* | A* | A* | 5 |
| Hutcheson et al. (2014) | A* | B | A* | A* | A** | B* | A* | A* | 8 |

^†^Selection:

(1) Representativeness of the exposed cohort: A, truly representative; B, somewhat representative; C, selected group; D, no description of the derivation of the cohort.

(2) Selection of the non-exposed cohort: A, drawn from the same community as the exposed cohort; B, drawn from a different source; C, no description of the derivation of the non-exposed cohort.

(3) Ascertainment of exposure: A, secure record; B, structured interview; C, written self-report; D, no description.

(4) For demonstration that the outcome of interest was not present at start of study: A, yes; B, no.

^‡^Comparability: For comparability of cohorts on the basis of the design or analysis: A, study controls for osteonecrosis of the jaw; B, study controls for any additional factor (e.g., age and severity of illness); C, not done.

^††^Outcome:

(1) Assessment of outcome: A, independent blind assessment; B, record linkage; C, self-report; D, no description.

(2) Was follow-up long enough for outcomes to occur? A, yes, (i.e. in-hospital or up to 30 d); B, no.

(3) Adequacy of follow-up of cohorts: A, complete follow-up and all subjects accounted for; B, subjects lost to follow-up was unlikely to introduce bias, because a small number were lost or a description was provided of those lost; C, follow-up rate 90% or lower and no description of those lost; D, no statement.

Table 2b. NOS for the Case-Control Study included.

|  | Selection ^†^ | | | |  | Exposure^††^ | | | |
| --- | --- | --- | --- | --- | --- | --- | --- | --- | --- |
| Included Studies | Adequacy of Case Definition | Representativeness of the Cases | Selection of Controls | Definition of Controls | Comparability^‡^ | Ascertainment of Exposure | Same Method of Ascertainment for Cases and Controls | Non-Response Rate | Total No. of stars |
| Kim ad al. (2018) | A* | A* | B | B | A* | A* | A* | A* | 6 |

^†^Selection

(1) Is this case definition adequate? A, yes, with independent validation; B, yes, eg record linkage or based on self reports; C, no description.

(2) Representativeness of the cases: A, Consecutive or obviously representative series of cases; B, Potential for selection biases or not stated.

(3) Selection of controls: A, Community controls; B, Hospital controls; C, No description.

(4) Definition of controls: A, No history of disease; B, No description of source.

^‡^Comparability: Comparability of cases and controls on the basis of the design or analysis: A, study controls for osteonecrosis of the jaw; B, study controls for any additional factor (e.g., age and severity of illness).

^††^Exposure:

(1) Ascertainment of exposure: A, Secured records; B, Structured interview where blind to case/control status; C, Interview not blinded to case/control status; D, written self-report or medical record only.

(2) Same method of ascertainment for cases and controls; A, yes; B, no.

(3) Non-response rate: A, Same for both groups; B, Non-respondents described; C, Rate different and no designation.
