# Supplementary material for: Identifying a predictive level of serum C-terminal telopeptide associated with a low risk of medication-related osteonecrosis of the jaw secondary to oral surgery: A systematic review and meta-analysis
Source: PLoS One. 2025 May 5;20(5):e0318260. doi: 10.1371/journal.pone.0318260 (PMC12052178; doi:10.1371/journal.pone.0318260)
Supplement: S1 File — (DOCX) [file pone.0318260.s003.docx]

Supplement 1: Electronic Search Strategy

PubMed: 96 results

(Bisphosphonate OR Denusomab) AND ("Bisphosphonate-Associated Osteonecrosis of the Jaw"[Mesh] OR "Osteonecrosis"[Mesh] OR (osteonecrosis AND jaw) OR BRONJ OR MRONJ) AND CTX OR ((c telopeptide OR C terminal) AND cross-link AND type 1 AND collagen))

EMBase: 150 results

(bisphosphonate OR denusomab) AND ('bisphosphonate-associated osteonecrosis of the jaw' OR 'osteonecrosis' OR (osteonecrosis AND jaw) OR mronj) AND CTX OR ((c AND telopeptide OR c) AND terminal AND 'cross link' AND type AND 1 AND collagen)

Cochrane Controlled Register of Trials: 4 results

(Osteonecrosis of the jaw) and (CTX) and (oral surgery)

One author (C.G.) examined the title and abstracts of all identified records and removed duplicates and obviously irrelevant reports. Two authors (C.G. and T.P.) screened independently the remaining references in full text. Any disagreements between the authors were discussed to reach consensus, and if consensus could not be reached, a third author (P.L.) was invited for the final decision. Reference lists were reviewed to identify additional relevant studies.

We applied the following exclusion criteria in a sequential order: (1) duplicates (between 2 electronic databases, or in a same electronic database but between 2 different journals), (2) language not English, (3) off topic, (4) population (no MRONJ or sCTX not performed or no oral surgery).
