# Supplementary material for: Identifying a predictive level of serum C-terminal telopeptide associated with a low risk of medication-related osteonecrosis of the jaw secondary to oral surgery: A systematic review and meta-analysis
Source: PLoS One. 2025 May 5;20(5):e0318260. doi: 10.1371/journal.pone.0318260 (PMC12052178; doi:10.1371/journal.pone.0318260)
Supplement: Attachment — Submitted filename: 2024 1020 Response to reviewers.docx [file pone.0318260.s006.docx]

| Questions/ Remarks | Response | Localisation in the manuscript |
| --- | --- | --- |
| 1) Did the authors assess for other risk factors that may affect serum CTX levels and the occurrence of MRONJ, such as comorbidities and length of bisphosphonate use? | First and foremost, we would like to thank you for all your constructive feedback, which has helped improve our manuscript.  We initially planned to collect all known risk factors for MRONJ. However, the only risk factors that we have been able to collect and analyse were the route of drug administration and the treatment indication (osteoporosis versus cancer). We specified this in the discussion chapter ““The various studies included in the meta-analysis reported little to no information on known established risk factors, such as smoking or oral hygiene,…” | Page 17 |
| 2) Can the authors describe more about how they derived the patients included in the analysis of individual data, what cases were excluded and included, and whether it was 58 or 38 cases? It is confusing to read on page 11, paragraph 12, that Individual data were available for 58 patients with MRONJ after oral surgery, but then in the caption for figure 4, just below that paragraph, n=38. | We revised the text to improve clarity." The second meta-analysis was conducted within the population with individual data available (n = 58) and was restricted to the population of patients treated with oral bisphosphonates for osteoporosis who developed MRONJ (n = 38).” | Page 12 |
| 3) In the discussion, an the authors discuss the possibility of other biomarkers that could be predictive of MR ONJ besides serum CTX, which is the reason why serum CTX is being particularly studied? | We chose to study sCTX because it is by far the most commonly reported biomarker in publications addressing the risk of MRONJ, and there would not have been sufficient data available for analysis with other biomarkers. Additionally, we selected sCTX because it is the most readily available and routinely used marker in clinical practice. |  |
| 4) Can the authors include citations that serum CTX measurement may not be reliable and can be variable depending on the time of day the sample was obtained and how it was measured? | Citations were added | Page 16-17 |
| While 260 is a relatively reasonable number based on this study, due to various limitations, such as the large variability of the studies and the possibility of bias, I think the authors should acknowledge the limitations more, especially in regard to the variability of assays, measurement, etc. More studies are needed to determine if 260 is an appropriate threshold. | We have indeed emphasized the need for caution in reading results and in predicting a threshold. However, with regard to CTX levels in patients on oral bisphosphonates treated for osteoporosis, it can be seen that less than 1% of these patients have a CTX level >260 pg/mL, regardless of the assay method used or the time of sampling, and are therefore at risk of developing osteonecrosis of the jaw.  “The findings from the individual data analysis require careful interpretation. These data were pooled from studies employing varying methodologies, such as differing inclusion criteria, sCTX collection times, and assay techniques. CTX levels may not have been consistently measured at the same time, which could introduce variability due to the circadian rhythm's influence on sampling. Even if it is recommended that blood samples should be taken in the morning, none of the studies reported the timing of sampling or whether the patients were fasting. There was also a lack of standardization in the methodologies used across different laboratories (47–50).  Additionally, variations were noted in the timing of sCTX measurements, with some taken preoperatively and others at the time of BRONJ diagnosis.” | Page 16 |
| For the results in table 1, why do the authors think IV bisphosphonates have a higher level of weighted average mean of CTX than oral bisphosphonates. Theoretically it should be the other way around. Was IV mainly used in cancer patients? | Indeed, IV bisphosphonates were used exclusively for cancer patients. |  |
| Can the authors put their findings in the context of recent review that states that we do not have reliable boneturnover markers to predict risk of MRONJ |  | Page 17 |
| Title Since the "safety threshold" was not directly established in this study, referring to the "predictive sCTX level" would be more appropriate. The short title is quite general and lacks specific information. It would be beneficial to make it more informative to better convey the study's focus. | I would like to modify the title as you asked but we are unfortunately constrained by the number of characters imposed by the revue It is stated that the title must not exceed 250 characters. Also the editor asked us to be more informative and add the notion that it is a systematic review and meta-analysis.  If it was possible we would like to write this title: Identifying a Threshold of Serum C-terminal telopeptide to Prevent Medication-Related Osteonecrosis of the Jaw: A systematic review and meta-analysis | Page 1 |
| “Among those treated with oral bisphosphonates for osteoporosis (n = 58),……” The data from Table 2 shows the number of MRONJ patients having bisphosphonate for osteoporosis is 38, not 58, is this number correct? | You are totally right, it is a typo, thank you for pointing it out. | Page 2 |
| This study did not provide sCTX levels for patients who did not develop MRONJ, making it impossible to establish the proposed sCTX level as a definitive threshold. Without this comparison, it remains unclear whether patients who have sCTX levels higher than the proposed threshold will not develop MRONJ. | We agree with you that it would have been preferable to have the sCTX value of the control group in order to be able to conclude on a minimum risk threshold. Unfortunately, we did not have the individual CTX values of the comparator group, i.e. patients on bisphosphonates who did not develop osteonecrosis. In fact, on a wider review of the literature, even in clinical trials this value is not always available. Nevertheless, given the rarity of this event, it is still interesting to have the CTX values of people who develop osteonecrosis in order to be able to deduce the minimum risk threshold above which osteonecrosis rarely occurs. |  |
| To enhance the article's impact, it is crucial to add more issues of MRONJ | It is a serious adverse event leading to several clinical issues: pain and discomfort, difficulty in eating and speaking, infection and fractures of the jaw. The chronic pain, functional impairments, and visible bone exposure can lead to significant impact on quality of life. | Page 3 |
| “The 1264 patients analyzed had all undergone oral surgery. They were treated with bisphosphonates, primarily for osteoporosis (96.3%)” Is this data from the same analysis as those in Table 1? | Yes, it is the same analysis.  The weighted average of mean and median sCTX according to route of administration and bisphosphonate indication of the patients with a MRONJ following oral surgery are only available for 1190 and missing for 74 patients. | Page 8 |
| “Table 1. Weighted average of mean and median sCTX according to route of administration and bisphosphonate indication of the patients with a MRONJ following oral surgery.” I noticed that your article states the number of patients who developed MRONJ following oral surgery was 113, not 1,190. Please verify the accuracy of the data in this table and ensure clarity. | We have modified the title of Table 1 “Weighted average of mean and median sCTX according to route of administration and bisphosphonate indication ~~of the patients with a MRONJ following oral surgery”~~ | Page 9 |
| To make the data more informative, the mean and median sCTX levels for MRONJ and non-MRONJ patients should be reported separately rather than as an overall combined level. | Unfortunately, of the 4 studies that presented a comparator group, none had individual CTX data and only two had mean sCTX. |  |
| - To make your discussion more compelling, discuss the strengths and weaknesses of meta-analysis and individual data analysis. This will demonstrate why the sCTX level from individual data analysis was proposed as the threshold level. | “Meta-analysis is a powerful tool for synthesizing evidence from multiple studies, providing increased statistical power, but it is constrained by the quality and heterogeneity of the included studies. Therefore, we conducted individual data analyses when possible, as this approach provides more granular, tailored insights with direct control over the data “ | Page 17 |
| The summary stating “our results suggest that in patients treated with bisphosphonates for osteoporosis, a sCTX threshold above 260 pg/mL is associated with minimal risk of MRONJ following oral surgery” cannot be conclusively drawn. This is because the sCTX levels of patients who did not develop MRONJ after oral surgery were not investigated. Therefore, it remains uncertain whether the sCTX levels of non-MRONJ patients are higher or lower than the proposed threshold | “These results need to be confirmed, particularly with sCTX data from a population of control patients.” | Page 17 |
